# Current achievements and future directions in genetic engineering of European plum (*Prunus domestica* L.)

**DOI:** 10.1007/s11248-018-0072-3

**Published:** 2018-04-12

**Authors:** Cesar Petri, Nuria Alburquerque, Mohamed Faize, Ralph Scorza, Chris Dardick

**Affiliations:** 1 Departamento de Producción Vegetal, Instituto de Biotecnología Vegetal, UPCT, Campus Muralla del Mar, 30202 Cartagena, Murcia Spain; 2 Departamento de Mejora Vegetal, CEBAS-CSIC, Campus de Espinardo, 30100 Espinardo, Murcia Spain; 3 Laboratory of Plant Biotechnology, Ecology and Ecosystem Valorization, Faculty of Sciences, University Chouaib Doukkali, 24000 El Jadida, Morocco; 4 Ag Biotech and Plant Breeding Consulting Services, Ralph Scorza LLC, Shepherdstown, WV 25443 USA; 5 USDA-ARS, Appalachian Fruit Research Station, 2217 Wiltshire Road, Kearneysville, WV 25430 USA

**Keywords:** Biotechnology, Woody plants, *Rosaceae*, Stone fruit, Plant breeding

## Abstract

In most woody fruit species, transformation and regeneration are difficult. However, European plum (*Prunus domestica*) has been shown to be amenable to genetic improvement technologies from classical hybridization, to genetic engineering, to rapid cycle crop breeding (‘FasTrack’ breeding). Since the first report on European plum transformation with marker genes in the early 90 s, numerous manuscripts have been published reporting the generation of new clones with agronomically interesting traits, such as pests, diseases and/or abiotic stress resistance, shorter juvenile period, dwarfing, continuous flowering, etc. This review focuses on the main advances in genetic transformation of European plum achieved to date, and the lines of work that are converting genetic engineering into a contemporary breeding tool for this species.

## Introduction

Plums (*Prunus domestica* and *P. salicina*) are second only to peach and nectarine in world stone fruit production reaching around 11 million tons a year and, according to FAO data, the gross product value in 2014 reached more than 9500 million USD (FAOSTAT 2017). The most important commercial cultivars belong to the hexaploid European plum (*Prunus domestica* L.) and the diploid Japanese plum (*P. salicina* L.). In addition, different plum species (e.g. *P. insititia*, *P. cerasifera*, *P. domestica*, and interspecific hybrids) are widely used as rootstocks for plum and other stone fruits. However, *P. domestica* has been the most important plum species historically.

Hybridization has been used to develop most of plums cultivars/clones, and along with the selection of clonal variants, it remains as the dominant technology. Sometimes, seedlings are the result of non-controlled pollination, but normally, hybridization crosses are controlled and parental individual/s are chosen and self- or cross-pollinated.

Conventional breeding of plum is constrained by their long reproductive cycle with long juvenile periods, complex reproductive biology and high degree of heterozygosity. Another drawback is the large land area necessary for planting seedling fruit tree populations and the associated expenses of field operations. Frequently, in order to obtain a new offspring that meets the desired agronomic and commercial characteristics it is necessary to perform several rounds of introgressive backcrossing (Petri and Scorza 2008). Since European plum average generation time is about 3–7 years, generally, 15–20 years are required from first fruiting to cultivar release.

Two are the main potential advantages of transformation for genetic improvement. Firstly, genetic engineering would allow the discrete modification of an established genotype; cultivar or rootstock. This process may require less time, labor and field space without the need of sexual crosses. Once a useful new transgenic clone is obtained, vegetative propagation through graftage or rooting of cuttings or micropropagation provides unlimited production of the desired clone, same way as any other conventional scion/rootstock. This would be an ideal situation, but unfortunately, transformation protocols for most important genotypes in the majority of fruit tree species are not currently available. Secondly, and perhaps most significantly, transformation may provide for genetic improvements that would otherwise be impossible using traditional breeding. A clear example are the transgenic papaya varieties ‘Rainbow’ and ‘SunUp’ resistant to papaya ringspot virus (PRSV), the most devastating disease threatening papaya production worldwide (Gonsalves et al. 2009). There is not natural source of PRSV resistance in the papaya germplasm, and genetic transformation allowed the production of PRSV resistant cultivars fast enough to fight against an emergent disease in Hawaii during the 90 s.

In most woody fruit species, transformation and adventitious regeneration are difficult, with low efficiency and often limited to a few genotypes or to seed-derived tissues (Petri and Burgos 2005). European plum has been the most successful species among *Prunus* to transform. This review focuses on the main advances in genetic transformation of European plum achieved to date, and the lines of work that are converting genetic engineering into a contemporary breeding tool for this species.

### European plum breeding objectives

The objectives are clearly different in scion or rootstocks breeding programs.

Plum scion breeding programs are established by the market requirements and consumer demand for the fruit as well as the regional climatic conditions, soils, and pest/disease pressures. European plums may be eaten fresh, canned, dried or distilled into brandy. Each use requires different selection criteria in the breeding program. Main breeding goals include resistance to biotic and/or abiotic stress, chilling requirements, tree size, productivity and fruit quality traits (Callahan 2008; Neumüller 2011). Some traits, such as high productivity and fruit quality, are shared goals for any fruit tree species. However European plum breeding programs have some peculiarities. Since most of the European plums are cultivated in countries/regions with severe winters, cold hardiness and late blooming are major breeding objectives. Related to plum affecting diseases breeding efforts have been focused on: brown rot, caused by the fungus *Monilinia* spp.; bacterial canker, caused by *Pseudomonas syringae* van Hall; bacterial spot, caused by *Xanthomonas campestris* pv. *Pruni*; plum leaf scald, caused by the bacteria *Xylella fastidiosa*, and Sharka, the most important disease affecting stone fruits, caused by the *plum pox virus* (PPV).

Plum rootstocks are selected based on traits such as rootstock-scion compatibility, scion vigor control, tolerance to different soil conditions (salinity, pH, drought, etc.), lack of root suckers and resistance to soil diseases and insects (Gainza et al. 2015). Iron chlorosis that often occurs in calcareous soils is one of the most limiting factors in the production of *Prunus.* One of the major pests in stone fruit orchards worldwide are Root-knot nematode (RKN), therefore obtaining resistant rootstocks is a main goal. Moreover, in poorly drained and dense clayish soils, *Prunus* rootstocks are at risk of being affected by diverse soil related diseases such as crown gall (*Agrobacterium tumefaciens*), crown rot (*Phytophthora* spp.), bacterial canker (*P. syringae* pv. *syringae*), oak root rot fungus, *Armillaria mellea* and *Armillaria tabescens* (Gainza et al. 2015).

As we will show in this review, the improvement of some of these traits has been the objective of genetic engineering in *Prunus domestica*.

The search of molecular markers associated to agronomical interesting traits in *Prunus* has focused the efforts of many scientists worldwide. Marker-assisted selection (MAS) saves time and money in fruit tree crops breeding programs allowing the elimination of undesirable plants from progeny populations as early as at the seedling stage. There are not yet molecular markers for agronomic traits available in *Prunus domestica*, which could be applied in breeding programs, due to the highly polymorphic hexaploid genome of this species (Neumüller 2011). However, in rootstock breeding programs MAS is being routinely used for selection of root-knot nematode (RKN) resistance since years ago (Claverie et al. 2004; Dirlewanger et al. 2004; Lecouls et al. 2004).

### Genetic transformation of European plum

In most woody fruit species transformation and regeneration of commercial cultivars is not routine and this is the main technical barrier to the application of biotechnology to fruit trees. The technological bottleneck is the difficulty or inability to regenerate shoots in vitro from clonal explants. Selection strategies (selection pressure, timing of selection, selective agent, selective-marker gene, etc.) to identify and to isolate the transgenic cells also constitutes a key factor for the success in the regeneration of transgenic shoots.

There are few documents reporting regeneration of transgenic European plum plants from transformed somatic cells (Table 1), although in most cases, only marker genes were introduced into the plant genome (Mikhailov and Dolgov 2007; Yancheva et al. 2002; Sidorova et al. 2017), with few reports of modification of agronomically important traits (Escalettes et al. 1994; Dolgov et al. 2010). Procedures developed for one cultivar are often not suitable for other cultivars. Essentially to date, ‘Startovaya’ remains as the only European plum cultivar amenable for transformation with the procedures developed by Dr. Dolgov’s laboratory (Table 1).

**Table 1:** Transformation of *Prunus domestica*

Cultivar/clone	Technique	Genes	Explant	TE^a^ (%)	References
Damas de Tolouse	*Rhizobium rhizogenes*	T-DNA (*ipt*)	Shoots	0.0	Escalettes et al. (1994)
		T-DNA (*ipt*), *PPV*-*CP*			
Marianna (GF8-1)	*R. radiobacter*	*npt*II, *gus*	Leaves	–	
		*npt*II, *gus, PPV*-*CP, hpt*			
B70146	*R. radiobacter*	*npt*II, *gus, PRV*-*CP*	Hypocotyls	3.0	Scorza et al. (1995)
Quetsche	*R. radiobacter*	*npt*II, *gfp*	Leaves	0.8	Yancheva et al. (2002)
Kyustendilska sinya				2.7	
Bluebyrd	*R. radiobacter*	*nptII, gus*	Hypocotyls	0.4	Gonzalez Padilla et al. (2003)
		*nptII, PDV*-*CP*		1.4	
		*nptII,PNRSV*-*CP*		0.7	
		*nptII, gus, TomRSV*-*CP*		4.2	
		*nptII, gus, antisense ACCO*		2.0	
		*nptII, PPV*-*CP hairpin*		42.0	Petri et al. (2008)
		*nptII, PDS hairpin*		15.0	
		*nptII, GAFP*		–	Kalariya et al. (2011)
		*PPV*-*CP hairpin* (marker-free)		2.5	Petri et al. (2011)
		*nptII, MdKN1*		–	Srinivasan et al. (2011)
		*nptII MdKN2*		–	
		*nptII, gus, KNOX1*		–	
		*nptII, PtFT1*		105.7	Srinivasan et al. (2012)
		*nptII, PpeGID1c hairpin*		–	Hollender et al. (2016)
Startovaya	*R. radiobacter*	*nptII, gfp*	Leaves	0.2	Mikhailov and Dolgov (2007)
		*hpt, gfp*		2.2	
		*hpt, PPV*-*CP hairpin*		1.1	Dolgov et al. (2010)
		*pmi, gfp*		1.4	Sidorova et al. (2017)
Stanley	*R. radiobacter*	*npt*II, *gus*	Hypocotyls	3.3	Mante et al. (1991)
		*npt*II, *gus, PPV*-*CP*		1.2	Scorza et al. (1994)
		*nptII, PPV*-*CP hairpin*		–	Hily et al. (2007)
		*nptII, gus, GAFP*		–	Nagel et al. (2008)
		*hpt, gus*		5.0	Tian et al. (2009)
		*hpt, ihp*-*elF4E*		–	Wang et al. (2013b)
		*hpt, ihp*-*elF(iso)4E*		–	
		*nptII, UTR/P1 PPV hairpin*		–	García-Almodóvar et al. (2015)
				–	Monticelli et al. (2012)
		*nptII, PpeDRO1*		–	Guseman et al. (2017)
Claudia verde	*R. radiobacter*	*nptII,* cyt*sod,* cyt*apx*	Hypocotyls	39.0	Faize et al. (2013)
				–	Diaz-Vivancos et al. (2013, 2016)
		*pmi, gus*		2.0	Wang et al. (2013a)
		*nptII, PpSAP1*		–	Lloret et al. (2017)
		*nptII, iaa*-*ipt hairpin*		7.7	Alburquerque et al. (2017)

^a^Transformation efficiency. When authors reported several TE, depending on different factors, the best results are displayed in the table. When not indicated, could not be deduced from the information provided by the authors

While regeneration of shoots from clonal explants is problematic, the use of seed-derived tissues seems to reduce the genotype effect as these explants are generally more likely to produce shoots in vitro. This has been the case in European plum, where numerous successful results have been published with different genotypes using seed-derived tissues as the explant source (Table 1). Since embryo tissues are not somatic, transformation of seed-derived material is not an ideal system for improving plum scion cultivars. Nevertheless, these procedures are very useful to generate of new engineered rootstock varieties and to introduce novel genes into plum germplasm.

**Fig. 1:**
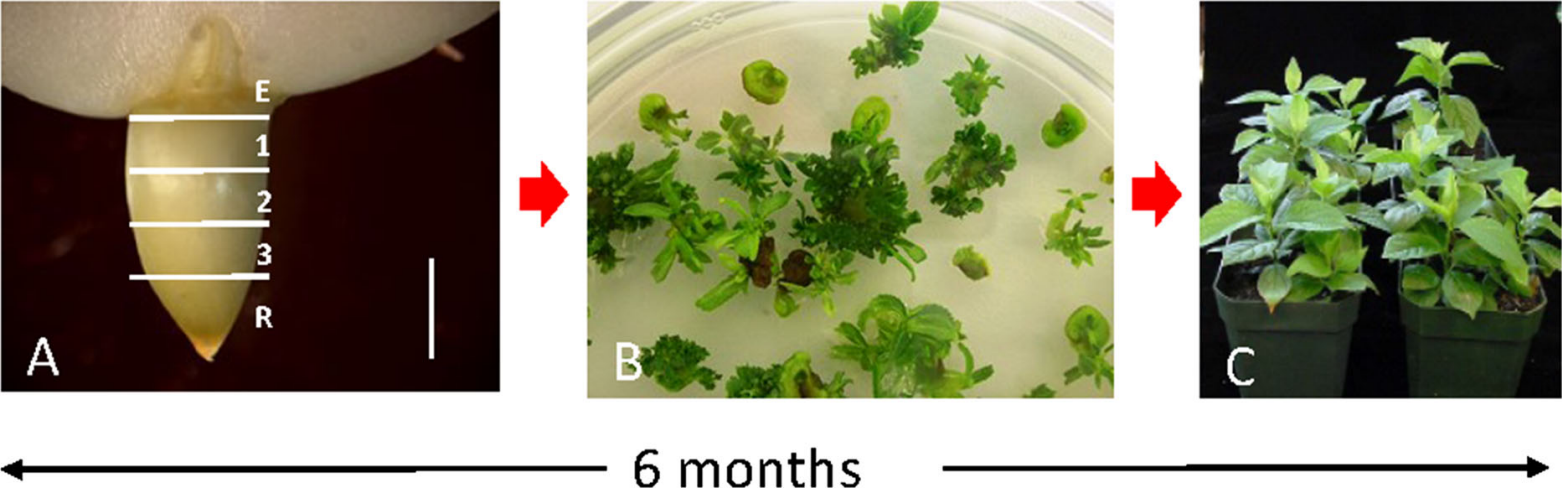
Regeneration of transgenic plums. **a** Source of explants: mature-seed hypocotyl slices. Epicotyl (E) and radicle (R) are not used. Vertical bar represents 1 mm. **b** Adventitious regeneration from hypocotyl slices in selective medium. **c** Transgenic plants cultured in a greenhouse

*Rhizobium radiobacter*-mediated (AKA *Agrobacterium tumefaciens*) transformation has been the principal technique applied to European plum (Table 1). In 1991, an initial transformation/regeneration protocol was described (Mante et al. 1991). This procedure used embryonic hypocotyl slices from mature seeds as the source of explants (Fig. 1a), and selection of transgenic plantlets was performed with kanamycin (km), using of *npt*II as the selectable marker gene (Mante et al. 1991). The protocol was later enhanced and currently has allowed transformation efficiencies up to 42% and enabled the production of self-rooted transgenic plants in the greenhouse in approximately 6 months (Fig. 1) (Gonzalez Padilla et al. 2003; Petri et al. 2008).

Both the original and improved protocol have been employed successfully for the introduction of agronomically useful genes into European plum (Alburquerque et al. 2017; Callahan and Scorza 2007; Diaz-Vivancos et al. 2013; Faize et al. 2013; García-Almodóvar et al. 2015; Gonzalez Padilla et al. 2003; Guseman et al. 2017; Hily et al. 2007; Hollender et al. 2016; Kalariya et al. 2011; Monticelli et al. 2012; Nagel et al. 2008; Petri et al. 2008, 2011; Scorza et al. 1994, 1995; Srinivasan et al. 2012; Wang et al. 2013b).

Normally in plant transformation, the transferred foreign DNA sequence(s) are stably incorporated into relatively few cells. Selectable marker genes are co-introduced with the gene(s) of interest and their function is the identification and/or selection of the transformed cells that are then induced to form shoots and whole transgenic plants. Once transgenic shoots are generated and stablished, selectable marker genes have no further purpose. At this point, their presence only creates complications with regulatory agencies and potential consumers. In this context, the high-throughput transformation system developed in European plum, allowed the regeneration of transgenic plums without the use of selectable marker genes (Petri et al. 2011).

Further procedures based on the plum hypocotyls as the source of explants with alternative selectable marker genes, other than *npt*II, have been published. These reports demonstrated the successful regeneration of transgenic European plum plants using hygromycin (*hpt* selective marker gene) or mannose (*pmi* selective marker gene) as the selective agents (Sidorova et al. 2017; Tian et al. 2009; Wang et al. 2013a, b). These systems may allow multiple genetic transformations of plum, and therefore, stacking several transgenic events in a single clone.

Similar methods, with hypocotyls slices as explants, have been applied successfully to Japanese plum (*Prunus salicina)* and apricot (*Prunus armeniaca*), showing the feasibility of the technique in another species, and the generation of transgenic plantlets from explants of different cultivars has been reported (Petri et al. 2015; Urtubia et al. 2008). Nevertheless, only marker genes were introduced into the plant genomes at that time.

### Agronomical traits genetically engineered in plum

#### Plum pox virus resistance

*Plum pox virus* (PPV) is the etiological agent of sharka disease. It causes chlorotic ring spots, vein clearing and leaf distortion. Symptoms recorded from fruits include severe fruit malformations, reduced fruit quality (Usenik et al. 2015), and premature abscission, reducing both yield and marketability (Sochor et al. 2012). Although eradication and quarantine programs are in place in most stone fruits producing countries, PPV is still widespread in all of them. The development of resistant clones appears as the most appropriate methodology to control of sharka disease (Sochor et al. 2012).

Ilardi and Tavazza (2015) reviewed the different biotechnological approaches applied to obtain PPV resistance in *Prunus*. Most of the transgenic strategies used are based on the heterologous expression of virus-derived sequences in plants. A notable example using this approach is the transgenic cultivar ‘Honeysweet’ developed at the USDA-AFRS (Kearneysville, WV, USA) (Scorza et al. 2013, 2016). In Abel et al. (1986), demonstrated that transgenic expression of a viral coat protein (CP) gene would prevent virus replication via inhibition of virus disassembly. This mechanism was dubbed CP-mediated protection and was subsequently used to engineer papaya, where the expression of papaya ringspot virus (PRSV) CP gene led to the generation of PRSV resistant papaya clones (Gonsalves 1998). Since PRSV-CP had significant homology to the PPV-CP gene, some experiments with the PRSV-CP were performed in transgenic plums. Protection against the virus was effective for several years in greenhouse tests, but after 32 months symptoms were evident and virus was detected throughout the plants (Scorza et al. 1995). The PPV-CP gene was then isolated, sequenced, and cloned (Ravelonandro et al. 1992) and used for *Agrobacterium*-mediated transformation of plum (Scorza et al. 1994). The regenerated transgenic plum lines were tested for resistance during 2 years under greenhouse conditions. The one transgenic clone (C5) that appeared highly resistant in the greenhouse tests did not express PPV-CP (Ravelonandro et al. 1997; Scorza et al. 2001). This clone “C5”, renamed as ‘HoneySweet’, showed low levels of transgene mRNA suggesting that resistance was not due to the expression of CP. In 1993, a new mechanism of virus resistance was reported whereby cells of plants transformed with a viral CP gene were resistant due to cellular degradation of viral RNA (Lindbo et al. 1993). This mechanism was later dubbed RNA interference (RNAi) (Fire et al. 1998). In ‘HoneySweet’, transgene methylation was observed along with the production of small interfering RNA (siRNA) specific to the PPV-CP transgene; indicating that resistance was through RNAi (Hily et al. 2004, 2005; Scorza et al. 2001). Constructs with self-complementary sequences separated by an intron produce “hairpin” RNA structures that efficiently cause the RNAi response. In the case of ‘Honeysweet’ resistance was not produced by an ihpRNA vector, but rather RNAi developed as a result of peculiarities of the insertion event that produced a hairpin of the PPV-CP transgene (Scorza et al. 2001).

The predicted rearranged PPV-CP (hairpin) sequence was further confirmed (Scorza et al. 2010), and the hairpin insert was cloned from C5 and expressed into ‘Bluebyrd’, demonstrating that the PPV-CP hairpin sequence from ‘HoneySweet’ plum provides PPV resistance (Scorza et al. 2010). Extensive testing and risk assessment, over 20 years, has shown that the resistance is highly effective, stable, durable, and environmentally safe (Scorza et al. 2013, 2016). ‘HoneySweet’ has successfully gone through the US regulatory process, that required critical safety evaluation by three agencies, the Animal and Plant Health Inspection Service (APHIS), Environmental Protection Agency (EPA) and the Food and Drug Administration (FDA), being the first woody perennial tree crop to have done so (Scorza et al. 2013). All the regulatory decision documents are available on line at the Center for Environmental Risk Assessment (CERA 2017). An important aspect in the deregulation of ‘Honeysweet’ is that new hybrids derived from its crosses will not require further regulatory approval.

‘HoneySweet’ is self-incompatible and sexually incompatible with most other *Prunus* species due to its hexaploidy. However, it has shown compatibility with several *P. domestica* plum varieties (Scorza et al. 2016). PPV-CP insert appears in heterozygosis, and therefore, PPV resistance segregates as a single dominant locus where approximately 50% of the progeny displays resistance to sharka disease (Scorza et al. 2016). ‘Honeysweet’ is currently being used as a source of PPV resistance in European plum breeding programs (Scorza et al. 2013).

Although, the clone has been patented (US PP15154 P2), it is freely available with no intellectual property restrictions for fruit production and for use as a source of PPV resistance in the USA (Scorza et al. 2016). Outside of the USA ‘HoneySweet’ has not received approval for cultivation yet, but the clone is being made freely available to researchers upon the certification that appropriate foreign regulatory approvals have been obtained (Scorza et al. 2016).

Since the development of ‘HoneySweet’, additional ihpRNA PPV-CP constructs were designed (Hily et al. 2007; Petri et al. 2008; Scorza et al. 2010) and additional PPV-CP silenced plum lines were generated and evaluated. Authors reported at that time that some of these new clones resulted resistant to PPV (Ravelonandro et al. 2013; Scorza et al. 2013).

In 2010, Dolgov and collaborators reported the generation of five additional independent transgenic PPV-CP ihpRNA plum lines. The novelty of this report lies in the source of the explants. In this case, the authors regenerated transgenic plantlets from clonal material (leaf segments) of the plum cultivar ‘Startovaya’. Authors reported at that time that the transgenic lines were under evaluation for PPV resistance (Dolgov et al. 2010). To our knowledge there is not further information published about these clones afterwards.

Hairpin RNA constructs targeting PPV sequences other than the PPV-CP have also been tested. A set of hairpin constructs targeting diverse regions on the 5′ end of the PPV genome were designed (Di Nicola-Negri et al. 2005). These constructs were based on the sequences of the most economically important viral isolates (PPV-D and PPV-M) and selecting highly conserved genomic regions. Among the vectors produced, promising results were obtained in *Nicotiana benthamiana* with the h-UTR/P1 RNAi, which encodes an ihpRNA containing the 50 nt untranslated region and a portion of *P1* gene of the Italian PPV-M ISPaVe44 isolate (Di Nicola-Negri et al. 2005, 2010). Later, transgenic *P. domestica* (cv. ‘Stanley’) seedling lines expressing the h-UTR/P1 construct were produced (García-Almodóvar et al. 2015; Monticelli et al. 2012). Transgenic clones were micrografted onto PPV infected ‘GF305’ and the presence of the virus in the grafted material was evaluated by RT-PCR. Seven out of ten clones (García-Almodóvar et al. 2015) and two out of two clones (Monticelli et al. 2012) displayed resistance to the PPV-D isolate.

Additional RNAi strategies in European plum have been reported targeting simultaneously different genome conserved regions among PPV isolates (Wang et al. 2015).

Ravelonandro et al. (2013) using computational target predictions produced artificial miRNA PPV silencing constructs. When engineered in *Nicotiana benthamiana* more than 70% of tested clones were resistant demonstrating the potential application of amiRNAs for the development of PPV resistant plums.

Other strategies to induce PPV resistance in plum have been based on targeting host factors. Plant viruses are obligate intracellular parasites and they require interaction with host factors for different steps in their cycle, such as translation, replication and/or movement. Consequently, knockout or knockdown of host genes essential for viral functions or mutations in essential host genes that impair their capacity to bind viral proteins can result in the loss of virus infectivity. In nature, such virus resistant traits usually occur as recessive characters. Some of these genes encode for eukaryotic translation initiation factors such as 4E (*eIF*4E), 4G (*eIF*4G) or their isoforms, *eIF(iso)*4E and *eIF(iso)*4G (Wang and Krishnaswamy 2012). The role of these translation initiation factors appears to be of particular importance for Potyvirus infection, but natural or induced resistance conferred by mutations/knock-out of these factors is not limited to the family *Potyviridae*, and can also target different (+) strand RNA viruses, such as carmoviruses, cucumoviruses, sobemoviruses and waikaviruses (Sanfaçon 2015).

The physical interaction between PPV-VPg and plum eIF(iso)4E was confirmed by Wang et al. (2013b) using a Y2H system. Moreover, a ihpRNA construct targeting the plum *eIF(iso)*4E was designed and introduced into plum. More than 80% of transgenic silenced *eIF(iso)4E* plums displayed resistance when challenged with the strain PPV-D (Wang et al. 2013b). If this transcription factor strategy is confirmed to be effective in field trials, it could be very interesting as it would lend itself to the engineering of disease resistance via gene editing techniques as well as provide a complementary mechanism to RNAi strategies. Additionally, transient *eIF(iso)*4E silencing in peach plants through virus induced gene silencing (VIGS) lead to PPV resistance up to 25 days post-inoculation (Cui and Wang 2017).

#### Fruit softening delayed plums

Fruit are harvested at a time determined by the handling properties that will yield the highest quality fruit that can withstand storage and transport. Reduced or delayed ethylene production in the fruit might result in a firmer fruit that could remain in the tree longer to develop more tree-ripened flavors, yet resist damage incurred during harvesting, processing and shipping (Callahan and Scorza 2007).

Plum hypocotyls (cv. ‘Blueblyrd’) were transformed with an antisense construct of a peach ACC oxidase (ACCO) gene (the enzyme responsible for the last step in ethylene synthesis) under the control of the CaMV35S constitutive promoter (Gonzalez Padilla et al. 2003). The antisense DNA strategy is similar to the RNAi approach. In this case, antisense mRNA anneals to the endogenous target mRNA. Consequently, dsRNA is formed and post-transcriptional gene silencing specific to the target sequence is triggered. Data analyses revealed that, in some transgenic lines, ethylene production and softening was delayed (Callahan and Scorza 2007).

#### Soil pathogens resistance

Several soil-borne organisms cause significant loses on *Prunus* production worldwide. Control of soil pathogens, such as fungi (*Phytophtora cinnamomi*, *Armillaria mellea*), bacteria (*Agrobacterium tumefaciens*) or nematodes (*Meloidogyne* sp.), is difficult once they are established in an orchard.

The Gastrodia antifungal protein (GAFP), a monocot mannose-binding lectin isolated from the Asiatic orchid *Gastrodia elata* (Hu et al. 1988), was expressed constitutively in *Nicotiana tabacum* and transgenic plants showed increased resistance against different soil pathogens (Cox et al. 2006). Later, *gafp* gene was engineered in transgenic plums (driven by the constitutive CaMV35S promoter or the polyubiquitin promoter *bul409*) and three transgenic lines exhibited increased tolerance to *Phytophthora root rot* (PRR), caused by *P. cinnamomi*, and to infection by *Meloidogyne incognita*. (Kalariya et al. 2011; Nagel et al. 2008). Authors stated these results as promising since the pathogen pressure in the experiments was much higher than the usual under natural field conditions, and long-term field trials will be necessary to confirm these results (Kalariya et al. 2011; Nagel et al. 2008).

Other studies have been focused on resistance to *Agrobacterium tumefaciens* through a biotechnological approach. Bacterial infection produces tumors in the plant known as crown galls, a disease that affects many perennial fruit, nut and ornamental crops, causing large annual losses to growers and nurseries world-wide (Alburquerque et al. 2012).

An RNAi approach, with a chimeric self-complementary construct, was designed to silence simultaneously the bacterium *ipt* and *iaaM* oncogenes. Its expression in *Nicotiana tabacum* induced resistance to crown gall disease (Alburquerque et al. 2012). Recently, this construct has been introduced and expressed in European plum, and several transgenic lines, derived from ‘Claudia verde’ hypocotyls, showed a significant reduction in the development of the crown gall disease after infection with the C58 and A281 *Agrobacterium* strains (Alburquerque et al. 2017).

#### Abiotic stress resistance

In *Prunus* spp., as an adaptation against the effects of cold and water stress, meristems go through dormancy during the cold period of autumn and winter. After transcriptomic analyses of differentially expressed transcripts during the dormancy process in reproductive buds of peach (*Prunus persica* [L.] Batsch), researchers identified a gene coding for a protein similar to Stress Associated Proteins (SAP) containing two specific Zn-finger domains (*PpSAP*) (Leida et al. 2010). Authors observed up-regulation in *PpSAP* expression in dormant buds and down-regulation occurred, along with dormancy release, and they stated that *PpSAP* expression seemed to be regulated with the developmental stage of buds under apparently variable environmental circumstances (Leida et al. 2010). *PpSAP* over-expression in transgenic plum plants led to alterations in leaf shape and increased tolerance to leaf desiccation, suggesting that this gene might be useful in manipulating abiotic stress tolerance in plants (Lloret et al. 2017).

Exposure to abiotic stress, such as salinity, deficit irrigation and osmotic stress is harmful for plants because of the induced damage caused by reactive oxygen species (ROS), such as hydrogen peroxide (H_2_O_2_) and superoxide radicals (O_2_^−^). To manage with ROS toxicity, plants have developed anti-oxidant mechanisms, by partially suppressing ROS production, or through their scavenging by enzymatic defenses such as superoxide dismutase (SOD), ascorbate–glutathione (ASC–GSH) cycle enzymes, catalase (CAT) and peroxidases (POX) (Asada 1999; Noctor and Foyer 1998).

Genes encoding cytosolic antioxidants ascorbate peroxidase (*cytapx*) from spinach (*Spinacia oleracea*) and Cu/Zn-superoxide dismutase (*cytsod*) from pea (*Pisum sativum*) were genetically engineered and constitutively expressed in European plum. Several transgenic plantlets showed an enhanced tolerance to salt stress when challenged to 100 mM of NaCl. The enzymatic study showed that the increased tolerance was related to modulation of enzymatic antioxidants as well as enhancement of non-enzymatic antioxidants such as glutathione and ascorbate (Diaz-Vivancos et al. 2013). Furthermore, one transgenic line with elevated ascorbate peroxidase activity was tolerant to severe water stress, correlated with a tighter control of water-use efficiency and enhanced photosynthetic performance (Diaz-Vivancos et al. 2016). The clones developed by these researchers could be very useful as rootstocks in arid and semi-arid regions affected by salinity and/or drought.

Other studies showed that overexpression of peach DEEPER ROOTING 1 (PpeDRO1) in *Prunus domestica* led to deeper-rooting phenotypes (Guseman et al. 2017). Their data suggested a potential application for DRO1-related genes to alter root architecture for drought avoidance and improved resource use, and the transgenic clones obtained in this study might be useful as rootstocks under water stress conditions (Guseman et al. 2017).

#### Modified size and shape trees

Plant size and architecture are currently main goals in many fruit trees genetic breeding programs, and some researchers have focused their studies in genetic factors controlling tree size and shape. In the management of an orchard many of the regular practices are associated with plant size and/or architecture, such as grafting, pruning, spraying, harvesting, etc. Therefore, tree size is crucial for a proper orchard management, optimizing productivity, labor and, consequently, benefits.

Transgenic *Prunus domestica* plums silenced for the gibberellic acid receptor GID1c displayed a range of dwarf phenotypes, suggesting that a reduction in GID1c levels could be utilized to develop semi-dwarf trees (Hollender et al. 2016). To these authors, knocking down GID1c expression, through genetic engineering or gene editing, or by selecting trees with naturally reduced GID1c expression in a breeding program, maybe a useful strategy to obtained new dwarf or semi-dwarf plum cultivars or rootstocks (Hollender et al. 2016).

#### Early-continuous flowering trees and fast breeding of plums

The tree fruit industry is facing highly dynamic situations such as climate change, reductions in available labor, increasing environmental concerns leading to restrictions in the use of agrichemicals, changing consumer preferences and the spread of exotic pathogens and insect pests. To meet these challenges, breeding new adapted fruit cultivars is critical. For tree fruit crops, the main factors slowing the rate of new cultivar development is the long juvenile period, that is, the time between seed planting and fruiting (3–7 years in the case of European plum), the large land areas necessary for planting seedling fruit tree populations, and the associated expenses of field operations. Hybridization is dependent upon flowering which occurs only once each year, and is dependent upon sufficient chill in the winter, warmth in the spring. Moreover, flowering and fruit set are significantly affected by many environmental factors such as severe low winter temperatures, spring frosts, high spring temperatures, and rain during pollination season. Manipulation of cultural conditions can shorten the period of juvenility, nevertheless promoting flowering through biotechnology has arisen in the last years as a feasible solution to overcome these limitations (van Nocker and Gardiner 2014).

Plum trees transformed with Poplar *Flowering locus T1* (*PtFT1*) showed altered architecture, dormancy requirement, and continuous flowering (Srinivasan et al. 2012). In the greenhouse conditions, transgenic plants over expressing *PtFT1* flowered and produced fruits continuously in few months (from 1 to 10 months, depending on the transgenic line) (Srinivasan et al. 2012). This means a reduction in the generation cycle of plum from 3 to 7 years to less than 1 year.

**Fig. 2:**
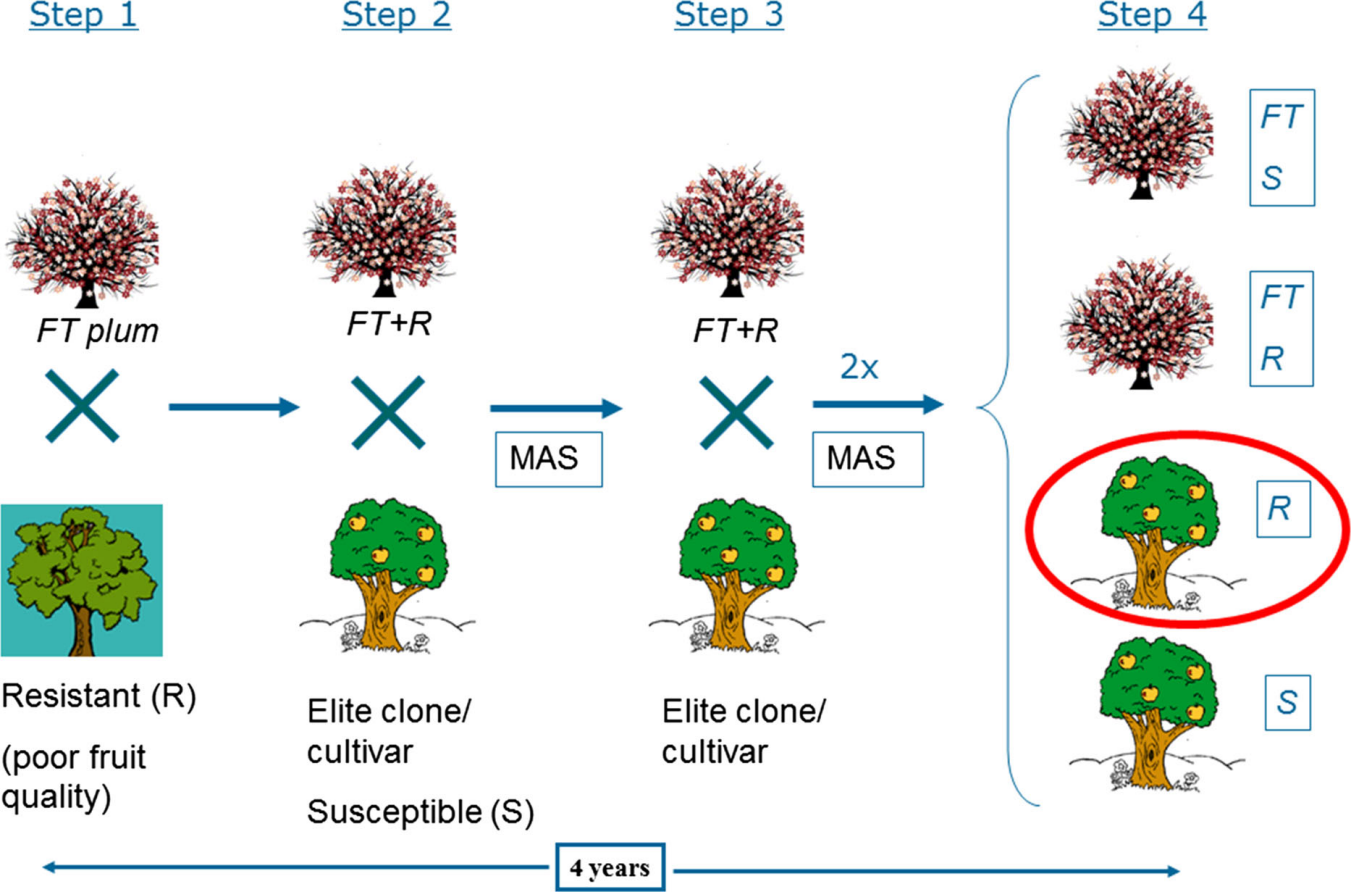
An example of FasTrack breeding technology. The scheme shows a procedure to move a disease resistant trait to an elite clone or commercial cultivar. *Step 1* Undertake initial cross of an early flowering FT-plum with a resistant genotype (R). Among the FT-plums progeny, select the resistant individuals using marker assisted selection (MAS). *Step 2* Undertake a cross of FT-resistant individuals with the desired type plum. *Step 3* Undertake two backcrosses with the original desired genotype using MAS to select the desirable traits. *Step 4* Select the resistant progeny (R) with the desired original type traits (in the red ellipse). These trees are non-transgenic and they can be field planted in evaluation plots. (Color figure online)

This unique phenotype displayed by the FT-plums is currently being applied in a novel breeding strategy. At the USDA-AFRS facility (Kearneysville, WV, USA), the FT-plums are being used in crosses for what they have termed “FasTrack” breeding (Fig. 2) (Scorza et al. 2014). Moreover, ‘FasTrack’ breeding is carried out in a greenhouse, therefore, the environmental limitations of winter chilling can be overcome, and flowering and fruit production is year-round. The system may allow for the rapid incorporation of important traits into plums.

The FT phenotype is dominant, therefore in the breeding process hemizygous early flowering progeny can be recurrently selected with the desired genotype and used for the next cross. Extreme dwarf plum plants are eliminated in each generation since they are not productive of flowers or fruit and therefore are not useful for breeding (R. Scorza, personal communication). When considerable improvements in the breeding process are clearly obvious, only seedlings that do not contain the early flowering gene are selected (Fig. 2). At this point, new plantlets are not genetically modified and can be planted in the field for their evaluation of agronomic and commercial traits. Afterwards, new clones could be used directly as new varieties or as elite lines for further breeding. More information about the innovative breeding program is available at http://ucanr.edu/sites/fastrack/ (accessed February, 2018).

In European plum ‘FasTrack’ breeding is in progress. ‘Prune d’Agen’ (also known as ‘Prune d’Ente or ‘French Prune’) along with its numerous clonal selections, is perhaps the most economically important *Prunus domestica* in the world. It accounts for most of the world’s trade in dried plums (prunes). The cultivar is very important in California, where it accounts for 99% of the dried plum production, however it is susceptible to sharka disease. Dried Plum industry members are especially concerned about this fact. Consequently, the ‘FasTrack’ system is currently being applied to move the PPV resistance trait from plum type ‘Honeysweet’ into ‘French’ type plums (http://ucanr.edu/sites/fastrack/DriedPlum/; accessed February, 2018). Additionally, the FT technology has been incorporated in a breeding program that attempt the obtainment of new stoneless plum cultivars (Callahan et al. 2015).

Similar rapid cycle crop breeding approaches, are currently being applied to other perennial tree fruits such as apple and citrus (Flachowsky et al. 2011; Le Roux et al. 2012; Rodriguez et al. 2014).

Furthermore, the FT-plums did not show chilling requirement and their phenotype was different from the wild type, losing apical dominance and showing a bushy or vine-like genotype (Srinivasan et al. 2012). For these authors, these new characteristics may facilitate the cultivation of European plum in tropical climates and the design of novel production methods for this crop, such as greenhouse intense production systems.

In fact, FT-plums have been proposed as a crop for spaceflights and extraterrestrial colonization (Graham et al. 2015). The small plant size and lack of any significant chilling requirement for flowering and fruiting are characteristics required for space travel and are not found in conventional fruit trees. In addition, beneficial effects of dried plums on bone health have been demonstrated (Deyhim et al. 2005; Franklin et al. 2006; Halloran et al. 2010; Rendina et al. 2013; Smith et al. 2014a, b), and therefore, FT-plums could be used as a countermeasure to microgravity-induced bone loss of the crewmembers during long term space missions (Graham et al. 2015).

### Future prospects in genetic engineering of plum

#### Gene editing

In recent years site-directed nucleases (SDNs), such as CRISPR/Cas9, have emerged as an attractive technology for production of mutated crops/cultivars (Pacher and Puchta 2017). Successful applications of CRISPR/Cas9 system to modify gene expression of several species, including perennial plants have been reviewed by Bortesi and Fischer (2015) and Samanta et al. (2016). In *Populus*, Fan et al. (2015) generated homozygous knock-out mutation in predicted loci in T0 generation with CRISPR/Cas9 system. Once again in *Populus*, Zhou et al. (2015) created knockout mutation in two genes involved in lignin and flavonoid biosynthesis achieving with 100% efficacy with this system.

Currently, the successful use of these strategies in woody perennial species is mainly limited to *Populus*, but there are many interesting traits that could potentially be modified by genome editing, including virus or insect resistance, herbicide tolerance, improved fruit quality traits, etc. However, there are some hindrances to the routine use of these new techniques in fruit trees, including their long life cycles and the obligate vegetative propagation. The combination of genome editing tools and the early flowering approach via transformation could allow the segregation of the induced mutation, the SDN used in the process, and the early flowering transgene in a relative short period. The new cultivars generated will not be transgenic, since the genetic engineering will only be used for inducing mutation and speeding up the breeding process. This might facilitate their deregulation and commercialization, making the process similar to any conventional new hybrid, and eliminating, or at least reducing, public concerns about biotech-crops. Good news in this direction are that USDA-APHIS does not consider breeding stock and cultivars produced from ‘FasTrack’ breeding as GM plants (https://www.aphis.usda.gov/biotechnology/downloads/reg_loi/Drs%20Scorza%20and%20Callahan%20Final.pdf).

In the application of sequence-specific nucleases, editing plant genomes without the stable incorporation of recombinant DNA into the target genome may help in the deregulation and commercialization process of new genetically modified cultivars or varieties. Therefore, DNA-free and/or virus-based genetic engineering tools may be of additional benefit in regions where legislation is less science-based. Viral vectors are being applied to express transiently the Cas9 protein and/or specific gRNAs (Reviewed by Pacher and Puchta 2017). In peach, a PRSV based-vector has been successfully used to silence endogenous genes through virus induced gene silencing (Cui and Wang 2017). This sort of vector could be used to deliver the CRISPR/Cas9 components transiently in European plum, as well as, DNA-based virus vectors, such as geminivirus, which have been successfully used for plant genome editing (Lozano-Durán 2016).

To date in plum, *eIF*(iso4E) appears as an remarkable target for SDNs since its down regulation displayed resistance to PPV infection in European plum and peach (Cui and Wang 2017; Wang et al. 2013b). The CRISPR/Cas9 technology was employed to introduce sequence-specific mutations at the *eIF(iso)*4E locus in *Arabidopsis thaliana* and complete resistance to *Turnip mosaic virus* (TuMV) was successfully engineered (Pyott et al. 2016). Transgene-free T2 generation was obtained by segregating the induced mutation from the CRISPR/Cas9 transgene.

Other plant translation factors have been identified as new possible targets for PPV resistance. In *Arabidopsis thaliana*, DNA-binding protein phosphatase 1 (AtDBP1) and a protein named GRF6, which interacts with both AtDBP1 and the mitogen-activated protein kinase 11, have been described as PPV susceptibility factors (Carrasco et al. 2014; Castello et al. 2010). If *Prunus* orthologues of these genes are identified at one point, they could be easily silenced following an RNAi approach in *Prunus domestica* and later evaluated to confirm the results observed in *Arabidopsis thaliana.* In addition, *Prunus persica* DEAD-box RNA helicase-like (PpDDXL) has been reported to interact with PPV-VPg and, PPV was not able to infect a knock-out mutant of *AtRH*8, an orthologue gene in *A. thaliana* (Huang et al. 2010). This protein seems to be a suitable candidate for the application of genome editing approaches since it is not involved in plant growth and development.

PPV resistance has also been shown to be affected by a cluster of six meprin and TRAF-C homology domain (MATHd) (Zuriaga et al. 2013). Mutations and deletions in the alleles were associated with the resistant phenotype. The mutated allele showed dominance and resistance was displayed in heterozygosity (Zuriaga et al. 2013), which makes this locus an excellent candidate as a target for future SDN approaches.

#### Genotype-independent procedures

As mentioned above, genotype is a key factor for transformation and procedures developed for specific genotypes are often not suitable for other cultivars. This fact is a big limiting factor in the application of genetic engineering to plum established commercial cultivars/clones, allowing discrete modifications in a desired genotype. Therefore, researchers have tried to overcome this problem with different strategies. Adventitious organogenesis and embryogenesis are usually very sensitive processes, strongly affected by minor changes. The use of regeneration-promoting genes could aid to reduce the genotype effect. Ectopic expression of the corn Knotted-like homeodomain (KNOX1) gene, which is involved in meristem and shoot apical meristem formation (Doerner 2003), enhanced the frequency of adventitious shoot regeneration from leaves of the ‘Bluebyrd’ plum cultivar from 0%, in the untransformed control explants, up to 96% (Srinivasan et al. 2011). Also, the *ipt* gene, oncogene involved in cytokines biosynthesis, increased regeneration of transformed plants from apricot in vitro leaf explants of the cultivar ‘Helena’ (López-Noguera et al. 2009). Further studies with additional cultivars need to be performed to probe the utility of these genes.

Transformation of meristematic tissues has been also proposed as a possible solution to reduce the genotype effect, allowing genetic manipulation of somatic cells of established cultivars. However, different authors have tried unsuccessfully to transform woody perennial plants meristems, where most of the transgenic regenerated shoots resulted as chimeras (Petri and Burgos 2005; Wang et al. 2009; Faize et al. 2010). Further studies would be necessary for the establishment of a dissociation methodology and regeneration of non-chimeric transgenic plants in order to make these methodologies applicable in woody perennial plants biotechnology.

## Conclusions

The European plum has been shown to be amenable to genetic improvement technologies from classical hybridization, to genetic engineering, to rapid cycle crop breeding (‘FasTrack’ breeding). *P. domestica* shows a high regeneration capacity from seed-derived explants and these regeneration rates allow the production of marker-free transgenic plants. Transformation from clonal material has also been demonstrated, although only in one cultivar (‘Startovaya’). A number of transgenic plums expressing resistance to virus, nematode, bacterial, and fungal diseases have been produced along with resistance to abiotic stresses and with altered fruit ripening have been produced in laboratories world-wide. Practical application of genetic engineering in *P. domestica* has been the development, U.S. regulatory approval, and release of a transgenic cultivar (‘HoneySweet’) with resistance to PPV, the most serious stone fruit virus disease. This release demonstrates the practical value of the application of these biotechnologies for plum, and for tree fruit improvement in general. Further, the *P. domestica* genetic engineering system along with the ‘FasTrack’ technology provides a platform for the rapid functional analysis of genes that not only affect vegetative characteristics, but also those affecting flowering, fruiting, and seed development. In turn, this genetic knowledge provides the raw material for that can be applied to produce further improvements to plum and other fruit crops that address the current and future challenges of fruit production. In the future, integration of classical and biotechnological technologies in breeding programs is crucial to obtain new improved plum trees, with good agronomical traits and fruit quality according to growers and consumers demands. Nowadays, the greatest barrier to achieve the transition to efficient, and effective breeding programs for plums, and all fruit tree crops, that will enable breeders to meet the demands of growers and consumers and enable the production of healthy, abundant crops in an era of changing climate is the lack of clear, efficient, science-based regulatory regimes that will allow for the application of modern methodologies in applied breeding.
